# Low-Light Image Enhancement Based on Constraint Low-Rank Approximation Retinex Model

**DOI:** 10.3390/s22166126

**Published:** 2022-08-16

**Authors:** Xuesong Li, Jianrun Shang, Wenhao Song, Jinyong Chen, Guisheng Zhang, Jinfeng Pan

**Affiliations:** School of Electrical and Electronic Engineering, Shandong University of Technology, Zibo 255000, China

**Keywords:** low-light image enhancement, image denoising, Retinex theory, low-rank approximation

## Abstract

Images captured in a low-light environment are strongly influenced by noise and low contrast, which is detrimental to tasks such as image recognition and object detection. Retinex-based approaches have been continuously explored for low-light enhancement. Nevertheless, Retinex decomposition is a highly ill-posed problem. The estimation of the decomposed components should be combined with proper constraints. Meanwhile, the noise mixed in the low-light image causes unpleasant visual effects. To address these problems, we propose a **C**onstraint **L**ow-Rank **A**pproximation **R**etinex model (CLAR). In this model, two exponential relative total variation constraints were imposed to ensure that the illumination is piece-wise smooth and that the reflectance component is piece-wise continuous. In addition, the low-rank prior was introduced to suppress the noise in the reflectance component. With a tailored separated alternating direction method of multipliers (ADMM) algorithm, the illumination and reflectance components were updated accurately. Experimental results on several public datasets verify the effectiveness of the proposed model subjectively and objectively.

## 1. Introduction

Images captured in low-light scenarios, i.e., in the darkness or night-time, not only lack favorable visual aesthetics but also suffer from noise and color distortions. This directly impedes the performance of many computer vision algorithms, e.g., image retrial and image recognition. With the boom and popularity of sensing technologies, the amount of images to be processed has skyrocketed, and the need for high-quality images in low-light conditions is of great urgency. Therefore, it is vital to construct an effective and practical method to deal with low-light image enhancement tasks.

There are various methods for low-light image enhancement, which can be divided into three categories, namely histogram equalization (HE)-based methods, Retinex-based methods, and deep-learning-based methods. HE is the property adjusted method that can enlighten dark images by extending the dynamic range of the image. However, this kind of method cannot adjust the detailed information of the image satisfactorily. Proposed by Land et al. [1], the Retinex theory can be regarded as a fundamental theory to human visual perception, and decomposes an image into illumination and reflectance components. It provides a robust and flexible framework for low-light image enhancement tasks [2]. Furthermore, the variational Retinex methods are utilized to estimate the piece-wise continuous reflectance component and piece-wise smooth illumination component [3]. Recently, low-light image enhancement methods based on deep learning have also been studied comprehensively. The efforts have been focused on exploring the learning-based methods after Bychkovsky’s et al. [4] pioneer work. The learning data-driven photo adjustment utilizes both the traditional machine learning techniques [5] and the deep neural networks [6,7]. However, the effectiveness of the learning-based methods highly depends on the massive amounts of high-quality labelled data, which is labor-intensive and time-consuming.

In this work, we propose a CLAR model for feasible low-light image enhancement. Since the Retinex decomposition is a highly ill-posed problem, we constructed two exponential relative total variation constraints for illumination and reflectance components, respectively. The two constraints enable the illumination to be piece-wise smooth, and the reflectance to be piece-wise continuous. However, the illumination and reflection components are interrelated and interactional during the loop iteration. To address this problem, an ADMM formulation was developed to optimize the CLAR model effectively. Meanwhile, excessive noise is inevitable in low-light images, and exists in the reflectance component [8]. To address this problem, the low-rank prior was introduced to suppress noise during the estimation of reflectance. Comparative experimental results demonstrate that the CLAR model can achieve a favorable performance in low-light image enhancement.

This paper is organized as follows: In Section 2, we briefly review the low-light image enhancement methods. In Section 3, the proposed model is elaborated. The experimental results are shown in Section 4. The conclusion of the work is demonstrated in Section 5.

## 2. Related Work

Histogram equalization (HE)-based methods generally utilize the histogram diagram to boost the contrast of the image. There are many variants of HE-based methods. The AHE [9] method calculates the local histogram and then reallocates the brightness to enhance the contrast of the image while preserving more details. However, the image is equally divided into several blocks, which will lead to the time complexity of the algorithm. The WTHE [10] algorithm can be applied to video enhancement and avoids the artifacts of over-enhancement and horizontal saturation effectively. However, the details are incomplete and noisy. The LDR [11] method expands the difference in adjacent pixels in the gray level through the layered difference representation of the 2D histogram, which has a considerable speed and enhancement effect. Nevertheless, the HE-based methods focus on contrast improvement while neglecting the effect of illumination conditions.

Learning-based methods model the feature maps from high visual quality images to enhance the low-light images. The pioneering supervised low-light enhancement method LLNet [12] lightens images with minimum pixel-level saturation by stacking a sparse denoising autoencoder. The MBLLEN method [13] decomposes the whole end-to-end network into a feature extraction module, enhancement module, and fusion module for low-light image enhancement, which can effectively suppress image noise and image artifacts in a low-light area. In addition, the Retinex theory is joined up with deep learning methods. According to the Retinex theory, the Retinex-Net [14] is composed of a decomposed network and an enhanced network. Specifically, the decomposed network aims to gain the reflectance and illumination components by decomposing the given image, and the enhanced network is built to enhance the estimated illumination. It overcomes the problem of the traditional method being limited by the reflectance and the capacity of the illumination decomposition model, but it depends on the two assumptions of Retinex theory decomposition. Similarly, KinD [7] introduces a degradation removal module in the estimation of the reflectance component. The light level can be flexibly adjusted according to the different needs of users, while effectively eliminating the visual defects amplified by enhancing dark areas. In addition to the aforementioned supervised learning methods, the unsupervised methods also find their way in the low-light image domain. EnlightenGAN [15], as the first unsupervised approach, employs a global–local discriminator to ensure the reality of the enhanced result. The main focus is on illumination enhancement without special attention to noise suppression, which may even be magnified in the enhanced image. Recently, Xiong et al. [16] proposed a two-stage GAN framework with pseudo-labelled samples. In this method, contrast enhancement and denoising are separated and good contrast enhancement and denoising results are obtained.

Retinex-based methods enhance low-light images by image decomposition. They postulate that the input low-light image *O* can be denoted as the product of the illumination *L* and the reflectance *R*, O=L⊙R [17]. The symbol ⊙ means the element-wise multiplication. The decomposed components can be converted back by dividing them alternatively, namely L=O⊘R and R=O⊘L, where ⊘ represents the element-wise division. Then, further processes to the decomposed components are utilized in order to obtain the enhanced result. As the pioneering methods in this domain, the single-scaled Retinex (SSR) [18] and multi-scaled Retinex (MSR) [19] were proposed for low-light image enhancement. The SSR is sensitive to high-frequency components and can better enhance the edge information in the image. However, the enhanced image looks unnatural and may be over-enhanced. Compared with SSR, MSR can realize color enhancement, color constancy, local dynamic range compression, and global dynamic range compression. The shortcoming of this method is that the edge is not sharp enough, and that high-frequency details cannot be improved significantly. The subsequent methods consider both the illumination and reflectance components in order to improve the performance [20,21]. However, estimating the illumination and reflectance components from a single image is an ill-posed problem [22]. To solve this issue, some researchers attempt to transform the Retinex decomposition into a statistical reasoning problem and solve the problem by imposing different constraints [23,24]. The variational Retinex methods commonly adopt the variational model to estimate the decomposed components [3]. The model is formulated as $\min_{L,R}{∥O-L⊙R∥}_{F}^{2}+N_{1}+N_{2}$, where $N_{1}$ and $N_{2}$ are regularized constraints for the decomposed components.

## 3. Methodology

### 3.1. Illumination and Reflectance Constraints

In Retinex theory, the key to preserving the brightness distribution consistency is to ensure that the illumination component *L* is piece-wise smooth [25]. In contrast, the reflectance component *R* should be piece-wise continuous while preserving the detail. To this end, it is necessary to impose appropriate constraints on the estimation of illumination and reflectance components. In the proposed model, we utilized the exponential relative total variation as the smoothness and continuous constraints by applying different exponential operations. The previous relative total variation method takes the relationship between the centered pixel and the neighbour pixels as the main consideration. It is composed of the window-based total variation and inherent variation [26]. The windowed total variation is formulated as

(1) $$P_{x/y}=\sum_{q\in R(p)}G_{\sigma }*\left|\nabla_{x/y}I_{q}\right|,$$

where the $P_{x}$ and $P_{y}$ are the windowed total variation in the centred pixel in vertical and horizontal directions. The windowed inherent variation $Q_{x}$ and $Q_{y}$ are defined as

(2) $$Q_{x/y}=\left|\sum_{q\in R(p)}G_{\sigma }*\nabla_{x/y}I_{q}\right|.$$

where *I* is the input image, $\nabla_{x/y}$ is a partial derivative in the horizontal or vertical direction and $G_{\sigma }$ is a Gaussian kernel with window size σ = 3. The symbol ∗ is a convolutional operator. R(p) is a rectangular region centered on the pixel *p*, and the pixel *q* belongs to R(p). The Retinex theory verifies the significant property that the gradient distributions of illumination and reflectance components are different. The gradient of the ideal piece-wise smooth illumination component tends to be small, whereas the gradient of the ideal reflectance component tends to be large. By applying exponents to the relative total variation, the constructed constraints can capture the gradient distribution characteristics of the decomposed components. The formulation of the illumination smooth constraint is formulated as

(3) $$S_{x/y}={\left(\frac{P_{x/y}\left(L\right)}{Q_{x/y}\left(L\right)+\epsilon }\right)}^{\gamma_{s}},$$

By combining the decay exponents, the detail preserving constraint is proposed. The formulation of the constraint is

(4) $$T_{x/y}=1⊘\left({\left|\frac{P_{x/y}\left(R\right)}{Q_{x/y}\left(R\right)+\epsilon }\right|}^{\gamma_{t}}+\varepsilon \right).$$

where ϵ = 0.001 and ε = 0.005. $\gamma_{s}$ and $\gamma_{t}$ are the exponential coefficients.

### 3.2. Nuclear Norm Minimization for Low-Rank Approximation

Noise commonly exists in low-light images due to the thermal noise in the electronic device and other factors. As prior knowledge, the rank of the noise-free images tends to be low. In contrast, the noisy image tends to have a high rank due to the chaotic distribution of the noise. The common solution to image denoising is the low-rank approximation method. Given the matrix *Y* as the input noisy image matrix, the main idea of low-rank approximation is to obtain a low-rank matrix *X* that is as close to the input *Y* matrix as possible. One way to achieve the low-rank matrix approximation is nuclear norm minimization (NNM).

Given a matrix *X*, the formulation of the nuclear norm is

(5) $${∥X∥}_{*}=\sum_{i}{\left|\sigma_{i}\left(X\right)\right|}_{1},$$

where $\sigma_{i}\left(X\right)$ means the *i*-th singular value of *X*. Furthermore, the optimization problem of NNM approximation with the Frobenius norm can be solved by a soft-thresholding operation on the singular values of the observed matrix [27]. The formulation of the optimization problem is

(6) $$\hat{X}=\arg\min_{X}{∥Y-X∥}_{F}^{2}+{∥X∥}_{*},$$

where $\hat{X}$ is the solution matrix.

### 3.3. Constraint Low-Rank Approximation Retinex Model

**Figure 1:**
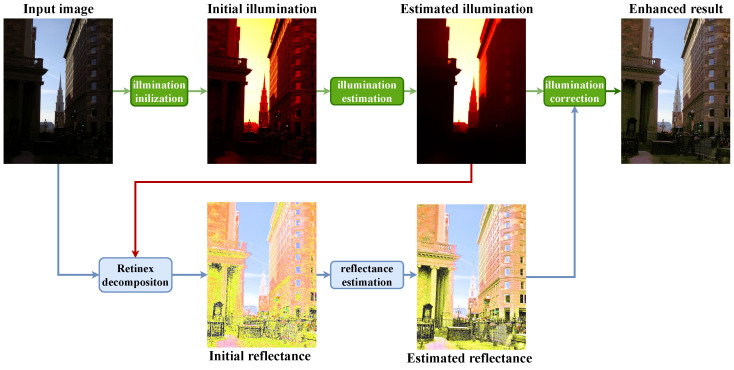
The framework of the proposed model. In the RGB space, the initial illumination component is first obtained to refine the estimated illumination component. Subsequently, the estimated illumination component is considered as the constant in order to obtain an initial reflectance component by Retinex decomposition. Then, the denoised reflectance component is estimated. Finally, the enhanced result is composed of the corrected illumination and the estimated reflectance.

The formulation of the constraint low-rank approximation Retinex model is formulated as

(7) $$\begin{array}{cc} \underset{R,L}{argmin}{∥R⊙L-O∥}_{F}^{2} & +\alpha \left(S_{x}{∥\nabla_{x}L∥}_{F}^{2}+S_{y}{∥\nabla_{y}L∥}_{F}^{2}\right)\\ \; & +\beta \left(T_{x}{∥\nabla_{x}R∥}_{F}^{2}+T_{y}{∥\nabla_{y}R∥}_{F}^{2}\right)+\sum_{i}{∥R_{i}\left(R\right)∥}_{*}, \end{array}$$

where α, β are the parameters that control the importance of different terms in the object function. ${∥O-L⊙R∥}_{F}^{2}$ constrains the fidelity between the observed image *O* and the reconstructed image L⊙R. The $S_{x}{∥{▽}_{x}L∥}_{F}^{2}$ and $S_{y}{∥{▽}_{y}L∥}_{F}^{2}$ enable the illumination map to be piece-wise smooth, and the $T_{x}{∥{▽}_{x}R∥}_{F}^{2}$ and $T_{y}{∥{▽}_{y}R∥}_{F}^{2}$ enable the reflectance map to be piece-wise continuous. $\sum_{i}{∥R_{i}\left(R\right)∥}_{*}$ is the nuclear norm term used to minimize the rank of the observed matrix of the *i*-th similar patch of reflectance component *R*. $R_{i}$ is a patch extraction operation. The framework of the proposed model is shown in Figure 1.

In the following parts, we elaborate the solutions to estimate the illumination component and reflectance component. These two components are divided into two separative problems and estimated independently.

### 3.4. Illumination and Reflectance Estimation Problems

#### 3.4.1. Illumination Estimation Problem

Picking all of the terms related to *L* in Equation (7), the formulation of the illumination estimation problem is

(8) $$\underset{L}{argmin}{∥R⊙L-O∥}_{F}^{2}+\alpha \left(S_{x}{∥\nabla_{x}L∥}_{F}^{2}+S_{y}{∥\nabla_{y}L∥}_{F}^{2}\right).$$

Estimating *L* from the reconstructed image may make the problem complicated and time-consuming. In addition, the influence of the reflectance component can be ignored when estimating the illumination component [22]. Therefore, the *L* is estimated from the initial illumination $\hat{L}$ with the proposed constraint in this model. The illumination estimation problem is reformulated as

(9) $$\underset{L}{argmin}{∥L-\hat{L}∥}_{F}^{2}+\alpha \left(S_{x}{∥\nabla_{x}L∥}_{F}^{2}+S_{y}{∥\nabla_{y}L∥}_{F}^{2}\right).$$

As proved by Guo et al. [28], the RGB image shares the same illumination component in three channels. The biggest value of the three channels is utilized as the initial illumination component $\hat{L}$, and the formulation of the $\hat{L}$ is

(10) $$\begin{array}{c} \hat{L}\left(x\right)=\max_{c\in \{R,G,B\}}L^{c}\left(x\right), \end{array}$$

where *x* is the pixel of the image. Furthermore, we constructed the augmented Lagrangian function to obtain the solution of the Equation (9), and the formulation is

(11) $$\begin{array}{cc} L(L,B,Z)= & ∥L-\hat{L}{∥}_{F}^{2}+\alpha \left(S_{x}{∥\nabla_{x}B∥}_{F}^{2}+S_{y}{∥\nabla_{y}B∥}_{F}^{2}\right)+Z\circ \left(B-L\right)\\ \; & +\frac{\mu }{2}{∥B-L∥}_{F}^{2},\\ \; & \;s.t.\;B=L, \end{array}$$

where *Z* is the Lagrangian multiplier and μ is a positive penalty scalar. By establishing the augmented Lagrange function, the number of variables to be iterated is increased to four, including *L*, *B*, *Z*, and μ. Then, Equation (9) is solved by using the alternating direction method of multipliers (ADMM) approach, which is commonly used to solve such convex problems. Therefore, each variable corresponds to a separate sub-problem and closed-form solution. The solutions of each sub-problems are given below:

**(1) Solution to*****L*****problem.** Collecting all of the terms related to *L* in Equation (11), the *L* problem is formulated as

(12) $$\begin{array}{c} \underset{L}{argmin}∥L-\hat{L}{∥}_{F}^{2}+Z\circ \left(B-L\right)+\frac{\mu }{2}{∥B-L∥}_{F}^{2}. \end{array}$$

To solve Equation (12), the matrix notation form of the function is rewritten as

(13) $$\begin{array}{cc} L^{\left(k+1\right)} & =\underset{L}{argmin}\left(L^{{\left(k\right)}^{T}}-{\hat{L}}^{T}\right)\left(L^{\left(k\right)}-\hat{L}\right)+Z^{\left(k\right)}\circ \left(B^{\left(k\right)}-L^{\left(k\right)}\right)\\ \; & +\frac{\mu^{\left(k\right)}}{2}\left(B^{{\left(k\right)}^{T}}-L^{{\left(k\right)}^{T}}\right)\left(B^{\left(k\right)}-L^{k}\right). \end{array}$$

Then, Equation (13) is differentiated and the derivative is set to 0. The solution to Equation (13) is formulated as

(14) $$2\left(L^{\left(k+1\right)}-\hat{L}\right)+Z^{\left(k\right)}\circ \left(B^{\left(k\right)}-L^{\left(k+1\right)}\right)+\mu^{\left(k\right)}\left(B^{\left(k\right)}-L^{\left(k+1\right)}\right)-Z^{\left(k\right)}=0,$$

(15) $$L^{\left(k+1\right)}=\frac{\mu B^{\left(k\right)}+Z^{\left(k\right)}+2\hat{L}}{(2+\mu^{\left(k\right)})I},$$

where *I* is the corresponding identity matrix.

**(2) Solution to*****B*****problem.** Collecting all terms related to *B* in Equation (11), we have:

(16) $$\begin{array}{cc} B & =\underset{B}{argmin}\alpha \left(S_{x}{∥\nabla_{x}B∥}_{F}^{2}+S_{y}{∥\nabla_{y}B∥}_{F}^{2}\right)+Z\circ \left(B-L\right)\\ & +\frac{\mu }{2}{∥B-L∥}_{F}^{2}. \end{array}$$

To solve Equation (16), the matrix notation form of the problem is written as

(17) $$\begin{array}{cc} B^{\left(k+1\right)} & =\underset{B}{argmin}\alpha \left(B^{{\left(k\right)}^{T}}D_{x}^{T}S_{x}D_{x}B^{\left(k\right)}+B^{{\left(k\right)}^{T}}D_{y}^{T}S_{y}D_{y}B^{\left(k\right)}\right)+Z^{\left(k\right)}\circ \left(B^{\left(k\right)}-L^{\left(k+1\right)}\right)\\ \; & +\frac{\mu }{2}\left(B^{{\left(k\right)}^{T}}-L^{{\left(k+1\right)}^{T}}\right)\left(B^{\left(k\right)}-L^{\left(k+1\right)}\right), \end{array}$$

where $D_{x}$ and $D_{y}$ are the Toeplitz matrices in horizontal and vertical directions, respectively. Then, Equation (17) is differentiated with respect to *B* and the derivative is set to 0; the solution to Equation (13) is formulated as

(18) $$\begin{array}{c} 2\alpha \left(D_{x}^{T}S_{x}D_{x}+D_{y}^{T}S_{y}D_{y}\right)B^{\left(k+1\right)}+Z^{k}+\mu^{\left(k\right)}\left(B^{k}-L^{k+1}\right)=0, \end{array}$$

(19) $$\begin{array}{c} B^{\left(k+1\right)}=\frac{\mu L^{\left(k+1\right)}-Z^{\left(k\right)}}{2\alpha \left(D_{x}^{T}S_{x}D_{x}+D_{y}^{T}S_{y}D_{y}\right)+\mu^{\left(k\right)}I}. \end{array}$$

**(3) Updating*****Z*****and μ.** The problem of updating *Z* and μ can be solved via

(20) $$\begin{array}{cc} Z^{\left(k+1\right)} & =Z^{\left(k\right)}+\mu^{\left(k\right)}\left(B^{\left(k+1\right)}-L^{\left(k+1\right)}\right)\\ \mu^{\left(k+1\right)} & =\mu^{\left(k\right)}\rho ,\;\rho >1 \end{array}$$

#### 3.4.2. Reflectance Estimation Problem

Collecting all of the terms related to *R* in Equation (7), the following formulation can be obtained:

(21) $$\underset{R}{argmin}{∥R⊙L-O∥}_{F}^{2}+\beta \left(T_{x}{∥\nabla_{x}R∥}_{F}^{2}+T_{y}{∥\nabla_{y}R∥}_{F}^{2}\right)+\sum_{i}{∥R_{i}\left(R\right)∥}_{*}.$$

Similar to the illumination estimation problem, the reflectance estimation problem is also solved by utilizing the ADMM algorithm. In addition, the *L* in Equation (21) is regarded as a constant after estimating the illumination component by solving the *L*-problem. The augmented Lagrangian function for Equation (21) is written as

(22) $$\begin{array}{cc} L(R,G,Y) & ={∥R⊙L-O∥}_{F}^{2}+\beta \left(T_{x}{∥\nabla_{x}G∥}_{F}^{2}+T_{y}{∥\nabla_{y}G∥}_{F}^{2}\right)+\sum_{i}{∥R_{i}\left(R\right)∥}_{*}\\ \; & +Y\circ \left(G-R\right)+\frac{\eta }{2}{∥G-R∥}_{F}^{2},\\ \; & \;s.t.\;G=R, \end{array}$$

where *Y* is the Lagrange multiplier and η is the positive penalty scalar. The *R* in the second term of Equation (21) is substituted by an auxiliary variable *G*. Then, the solutions to each sub-problem for the corresponding variable are as follows.

**(1) Solution to*****G*****problem.** Neglecting the terms unrelated to *G*, we have the following problem:

(23) $$\begin{array}{c} G=\underset{G}{argmin}\beta \left(T_{x}{∥\nabla_{x}G∥}_{F}^{2}+T_{y}{∥\nabla_{y}G∥}_{F}^{2}\right)+Y\circ \left(G-R\right)+\frac{\eta }{2}{∥G-R∥}_{F}^{2}. \end{array}$$

Similar to the solution to Equation (17), the matrix notation form of the problem is written as:

(24) $$\begin{array}{cc} G^{\left(k+1\right)} & =\underset{G}{argmin}\beta \left(G^{{\left(k\right)}^{T}}D_{x}^{T}T_{x}D_{x}G^{\left(k\right)}+G^{{\left(k\right)}^{T}}D_{y}^{T}T_{y}D_{y}G^{\left(k\right)}\right)\\ \; & +Y^{\left(k\right)}\circ \left(G^{\left(k\right)}-R^{\left(k\right)}\right)+\frac{\eta^{\left(k\right)}}{2}\left(G^{{\left(k\right)}^{T}}-R^{{\left(k\right)}^{T}}\right)\left(G^{\left(k\right)}-R^{\left(k\right)}\right) \end{array}$$

Then, Equation (24) is differentiated and the derivative is set to 0. The solution is formulated as

(25) $$\begin{array}{c} 2\beta \left(D_{x}^{T}T_{x}D_{x}+D_{y}^{T}T_{y}D_{y}\right)G^{\left(k+1\right)}+Y^{\left(k\right)}+\eta^{\left(k\right)}\left(G^{\left(k+1\right)}-R^{(k}\right)=0 \end{array}$$

(26) $$\begin{array}{c} G^{\left(k+1\right)}=\frac{\eta R^{\left(k\right)}-Y^{\left(k\right)}}{2\beta \left(D_{x}^{T}T_{x}D_{x}+D_{y}^{T}T_{y}D_{y}\right)+\eta^{\left(k\right)}I} \end{array}$$

**(2) Solution to*****R*****problem.** Collecting the terms related to *R*, the formulation of estimating the variable *R* is

(27) $$\begin{array}{c} R=\underset{R}{argmin}{∥R⊙L-O∥}_{F}^{2}+\sum_{i}{∥R_{i}\left(R\right)∥}_{*}+Y\circ \left(G-R\right)+\frac{\eta }{2}{∥G-R∥}_{F}^{2}. \end{array}$$

The $R_{i}\left(R\right)$ is the patch-level representation for the corresponding *R*. To simplify the problem, Equation (27) is reformulated as

(28) $$\begin{array}{cc} R= & \underset{R}{argmin}\sum_{i}{∥R_{i}\left(R\right)⊙L_{i}-O_{i}∥}_{F}^{2}+\sum_{i}{∥R_{i}\left(R\right)∥}_{*}\\ \; & +\sum_{i}Y_{i}\circ \left(G_{i}-R_{i}\left(R\right)\right)+\sum_{i}\frac{\eta }{2}{∥G_{i}-R_{i}\left(R\right)∥}_{F}^{2}, \end{array}$$

where $L_{i}$, $O_{i}$, $Y_{i}$, and $G_{i}$ are the *i*-th patch-level representations of *L*, *O*, *Y*, and *G*, respectively. For further simplicity, the problem is transformed into each *i*-th patch-level location. The operator with respect to *i* is omitted in the rest part. Then, Equation (28) is reformulated as

(29) $$\begin{array}{c} R=\underset{R}{argmin}{∥R⊙L-O∥}_{F}^{2}+{∥R∥}_{*}+Y\circ \left(G-R\right)+\frac{\eta }{2}{∥G-R∥}_{F}^{2}. \end{array}$$

Equation (29) is further modified to solve the problem. The reform can be formulated as

(30) $$\begin{array}{cc} \; & \underset{R}{argmin}{∥R-{\bar{R}}^{\left(k\right)}∥}_{F}^{2}+{∥R∥}_{*}^{2},\\ \; & {\bar{R}}^{\left(k\right)}=\frac{2O\circ L+\eta^{\left(k\right)}G^{\left(k+1\right)}+Y^{\left(k\right)}}{2L^{2}+\eta^{\left(k\right)}}. \end{array}$$

From then on, the original *R*-*R* problem is transformed into the standard low-rank minimization problem, which can be formulated as

(31) $$\begin{array}{c} R^{\left(k+1\right)}=US_{\tau }\left(\Sigma \right)V^{T}, \end{array}$$

where ${\bar{R}}^{\left(k\right)}=U\Sigma V^{T}$ is the singular value decomposition of the ${\bar{R}}^{\left(k\right)}$ and $S_{\tau }\left(\Sigma \right)$ is the soft-thresholding operation.

**(3) Updating*****Y*****and η.** The updating problem of *Y* and η can be solved via

(32) $$\begin{array}{cc} Y^{\left(k+1\right)} & =Y^{\left(k\right)}+\eta^{\left(k\right)}\left(G^{\left(k+1\right)}-R^{\left(k+1\right)}\right)\\ \eta^{\left(k+1\right)} & =\eta^{\left(k\right)}\rho ,\;\rho >1 \end{array}$$

### 3.5. Retinex Composition

Since the illumination and reflectance components have been estimated by solving the above problems, the final step is to adjust *L* to improve the visibility and brightness of the input image. Therefore, the gamma correction [29] is adopted as the illumination adjustment method. The corrected illumination $L_{Gamma}$ is written as

(33) $$L_{Gamma}=L^{\frac{1}{\gamma }}.$$

The final enhanced image $\hat{O}$ is generated by the composition of the estimated reflectance component and the corrected illumination component, which is formulated as

(34) $$\hat{O}=R⊙L^{\frac{1}{\gamma }},$$

where γ is empirically set to 2.2 [20,30].

## 4. Experimental Results and Analysis

### 4.1. Experiment Settings and Implementation Details

All of the experiments were performed on MATLAB R2019b with Intel i7-9700K CPU @3.60 GHz and 32 GB memory. We set the key parameters as $\gamma_{s}=1.25$, $\gamma_{t}=0.75$, α=0.015, and β=0.01. For a fair comparison, the results of the compared methods were reproduced by official codes. The proposed method was evaluated on six benchmarks, i.e., LIME [28], DICM [11], LOL [14], MEF [31], NPE [32], and VV (https://sites.google.com/site/vonikakis/datasets, accessed on 25 July 2022). Comparison analyses were carried out on ten competitors, including HE [33], SSR [18], MSRCR [34], CVC [35], Dong [36], LIME [28], Kindle [7], Jiep [37], ZERO-DCE [38], and ZERO-DCE++ [39].

### 4.2. Decomposition Analysis

**Figure 2:**
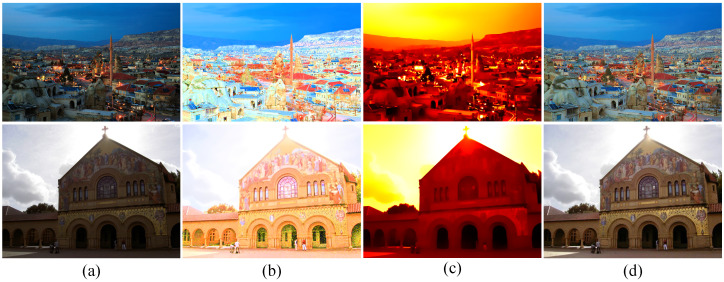
Visual evaluation of Retinex decomposition. (**a**) Input low-light image, (**b**) the estimated reflectance component, (**c**) the estimated illumination component, (**d**) the enhancement result.

The example results of the Retinex decomposition of the proposed model are shown in Figure 2. As mentioned above, the illumination map should be piece-wise smooth, and the reflectance should be piece-wise continuous. From the first row and second row in Figure 2b,c, the estimated reflectance and illumination component is preferable without noise increasing. Through the first row in Figure 2b, the details of buildings and hillsides are well-preserved in the reflectance component. As shown in the second row of Figure 2b, the murals on the wall and the patterns on the columns in the refined reflectance component are clear. As for the estimated illumination component, its spatial smoothness makes the global brightness of the image evenly distributed, e.g., the church in the second row of Figure 2b).

### 4.3. Subjective Visual Evaluation

**Figure 3:**
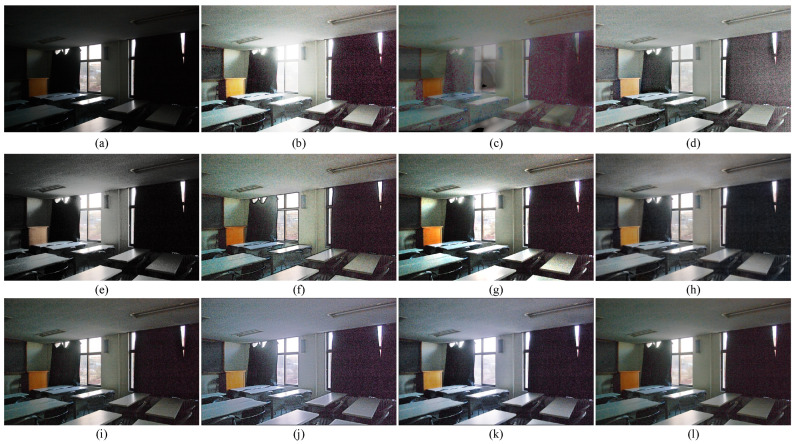
Visual comparison between low-light image enhancement results in the exemplar image. (**a**) Input (**b**) HE [33], (**c**) SSR [18], (**d**) MSRCR [34], (**e**) CVC [35], (**f**) Dong [36], (**g**) LIME [28], (**h**) Jiep [37], (**i**) Kindle [7], (**j**) ZERO-DCE [38], (**k**) ZERO-DCE++ [39], (**l**) ours.

**Figure 4:**
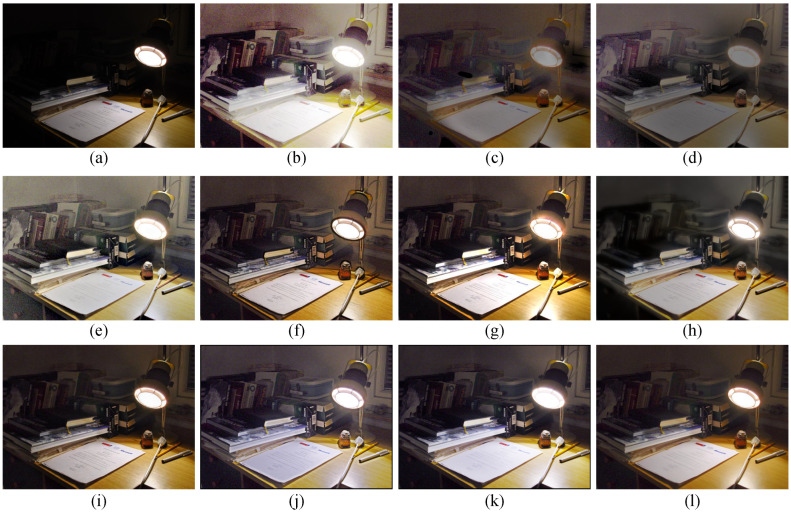
Visual comparison between low-light image enhancement results in the exemplar image. (**a**) Input (**b**) HE [33], (**c**) SSR [18], (**d**) MSRCR [34], (**e**) CVC [35], (**f**) Dong [36], (**g**) LIME [28], (**h**) Jiep [37], (**i**) Kindle [7], (**j**) ZERO-DCE [38], (**k**) ZERO-DCE++ [39], (**l**) ours.

**Figure 5:**
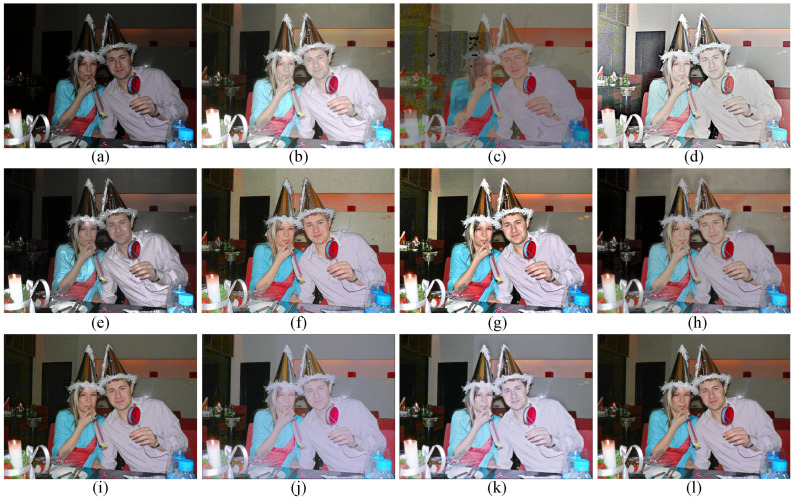
Visual comparison between low-light image enhancement results in the exemplar image. (**a**) Input (**b**) HE [33], (**c**) SSR [18], (**d**) MSRCR [34], (**e**) CVC [35], (**f**) Dong [36], (**g**) LIME [28], (**h**) Jiep [37], (**i**) Kindle [7], (**j**) ZERO-DCE [38], (**k**) ZERO-DCE++ [39], (**l**) ours.

**Figure 6:**
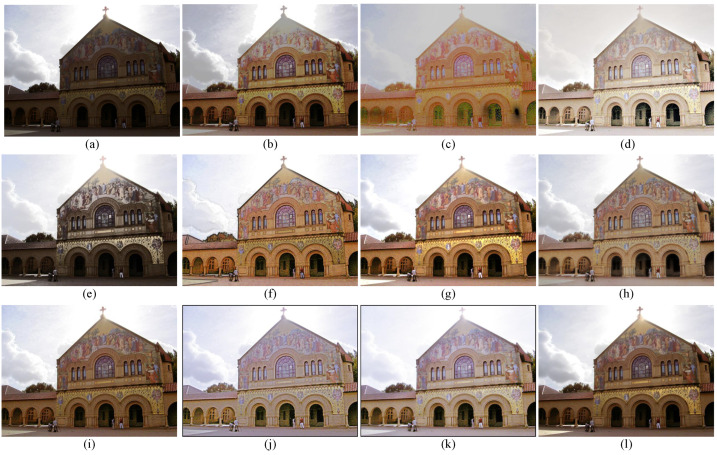
Visual comparison between low-light image enhancement results in the exemplar image. (**a**) Input (**b**) HE [33], (**c**) SSR [18], (**d**) MSRCR [34], (**e**) CVC [35], (**f**) Dong [36], (**g**) LIME [28], (**h**) Jiep [37], (**i**) Kindle [7], (**j**) ZERO-DCE [38], (**k**) ZERO-DCE++ [39], (**l**) ours.

To subjectively verify the enhancement performance between the proposed method and competitors, the comparative results are shown in Figure 3, Figure 4, Figure 5 and Figure 6. Some meaningful information can be observed from these figures.

In Figure 3, the input image suffers from darkness and unevenly distributed illumination. In Figure 3b, the method of HE [33] could brighten the low-light image, but the noise in the image is also amplified. In Figure 3c, the result generated by the SSR [18] suffers not only from the serious artifacts, but also from the strongly boosted noise. Even MSRCR [34] can improve the brightness of the image and maintain details, but the color distortion and noise amplification are serious in the results generated by MSRCR, e.g., Figure 3d. In Figure 3e, the CVC [35] fails to enhance the brightness of the image effectively. The edge texture of the desk tends to be overly thick in Figure 3f. The distinct halo appears in the bright area near the window in Figure 3g of the result generated by LIME [28]. In Figure 4b, the bright region around the reading lamp is also vague. In Figure 3h and Figure 4h, our method could not only enhance the dark areas with uneven illumination distribution but could also avoid the halo and noise amplification. In Figure 4h, the result generated by Kindle [7] tends to be detail-obscured, e.g., the books on the table. In Figure 6i, although the global brightness of the image can be enhanced by Jiep [37], the floral designs tend to be color-distorted. As for ZERO-DCE and ZERO-DCE++, the color and atmosphere of the enhanced images are changed, e.g., in Figure 5j,k.

Similarly, HE, SSR, and MSRCR amplify the noise in the background in Figure 5. Meanwhile, the result generated by CVC tends to be darker than others in Figure 5e. Observing the people in Figure 5, it is obvious that the Dong [36] and LIME [28] make the enhancement of the face and the clothes slightly theatrical. The enhancement result of our method is consistent with people’s visual perception in Figure 5h.

### 4.4. Quantitative Evaluation

**Figure 7:**
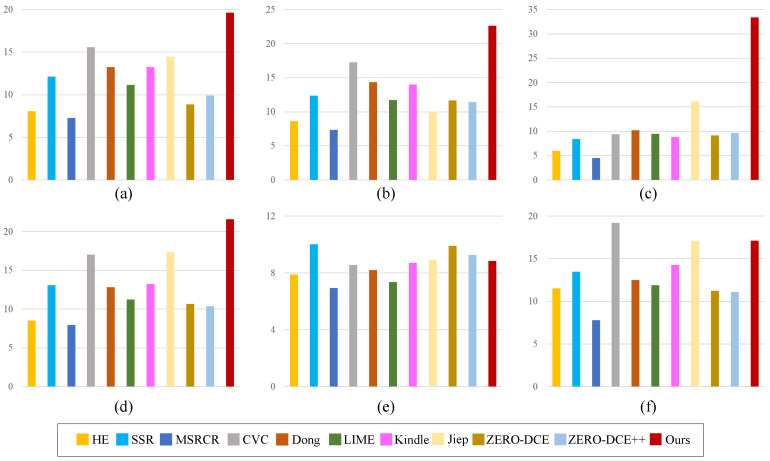
Quantitative comparisons in terms of PSNR. (**a**) LIME [28], (**b**) DICM [11], (**c**) LOL [14], (**d**) MEF [31], (**e**) NPE [32], (**f**) VV.

**Figure 8:**
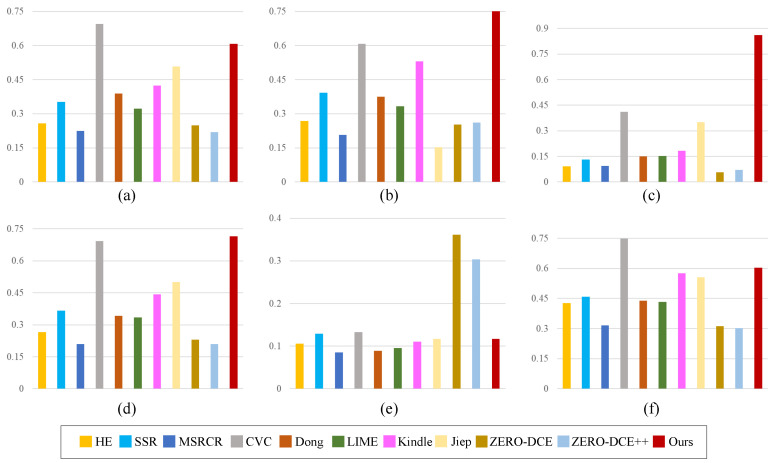
Quantitative comparisons in terms of SSIM. (**a**) LIME [28], (**b**) DICM [11], (**c**) LOL [14], (**d**) MEF [31], (**e**) NPE [32], (**f**) VV.

Apart from the visual evaluation comparisons, the quantitative comparisons were also utilized to verify the effectiveness of the proposed model. For image quality assessment, many metrics have been proposed [40]. For example, Zhai et al. [41] proposed the LIEQA to evaluate the image quality from four aspects, namely, luminance enhancement, color rendition, noise evaluation, and structure preserving. Zhou et al. [42] put forward the SRIF indicator to evaluate the visual quality of super-resolved images. Still, the peak signal noise ratio (PSNR) and structural similarity index measure (SSIM) [43] are mostly adopted metrics for objective evaluations in the domain of low-light image enhancement [44]. The quantitative comparative results on PSNR and SSIM are depicted in Figure 7 and Figure 8.

PSNR quantifies to what extent the image is affected by noise, approximating the human perception of the image. For PSNR, the bigger value represents the better image quality. SSIM quantifies a measure or prediction of image quality relative to the original uncompressed or undistorted image as a reference. For SSIM, the bigger value represents the better image quality. As demonstrated in Figure 7, our method outperforms the competitors on four datasets, namely, LIME [28], DICM [11], LOL [14], and MEF [31]. In addition, our method ranks in the top three on the VV dataset. From Figure 8, we can see that the proposed method ranks the first on three datasets, namely, DICM [11], LOL [14], and MEF [31]. Meanwhile, the proposed method ranks the second on LIME and VV datasets, which are approximate to the highest scores.

### 4.5. Denoising Evaluation

**Figure 9:**
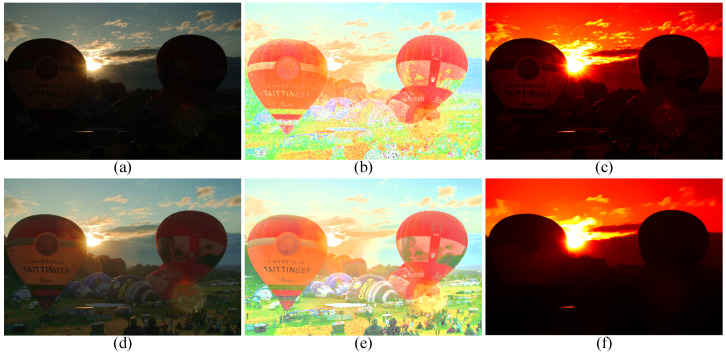
Visual evaluation of Retinex decomposition. (**a**) Original low-light image, (**b**) initial reflectance component $\hat{R}$, (**c**) initial illumination component $\hat{L}$, (**d**) corresponding enhancement result, (**e**) refined reflectance component *R*, (**f**) estimated illumination component *L*.

**Figure 10:**
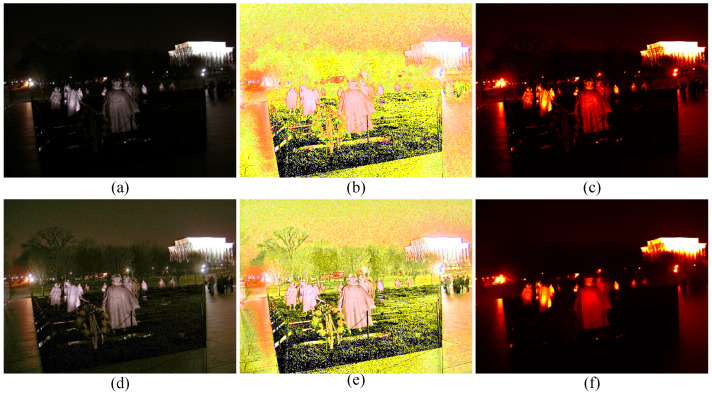
Visual evaluation of Retinex decomposition. (**a**) Original low-light image, (**b**) initial reflectance component $\hat{R}$, (**c**) initial illumination component $\hat{L}$, (**d**) corresponding enhancement result, (**e**) refined reflectance component *R*, (**f**) estimated illumination component *L*.

In addition to verifying the effectiveness of the enhancement performance, the denoising ability of our model can also be demonstrated in Figure 9 and Figure 10. For the realistic and intuitive visual perception, the illumination component is transformed into the hot map without gamma correction.

Through Figure 9b,c, it is shown that extracting the clean initial illumination map from a low-light image will leave most of the noise in the reflectance component. From Figure 9c–f, the results show that the illumination obtained by solving Equation (8) could be piece-wise smooth without noise amplification. Through Figure 9b,e and Figure 10b,e, the estimated reflectance component could contain less noise and enhance the contrast by comparing with the initial reflectance component. This proves the effectiveness of the low-rank approximation in our model. In general, our method could brighten the low-light image while avoiding noise amplification.

**Figure 11:**
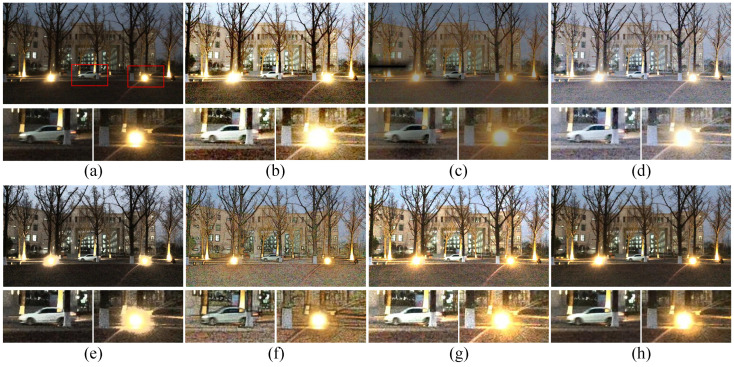
Visual comparison between enhancement results in a noisy low-light image. (**a**) Input, (**b**) HE [33], (**c**) SSR [18], (**d**) MSRCR [34], (**e**) CVC [35], (**f**) Dong [36], (**g**) LIME [28], (**h**) ours.

Furthermore, more comparative results of the denoising comparison for low-light image enhancement methods are depicted in Figure 11. Distinctly, the methods of SSR, CVC, and Dong fail to maintain the details while brightening the image. The results generated by HE and LIME tend to be over-enhanced, e.g., the edges of the light in Figure 11b,g are obscured. In addition, the method of MSRCR fails to maintain the color constancy while amplifying the noise. Compared to competitors, our method could generate a preferable enhancement result.

### 4.6. Ablation Study

**Figure 12:**
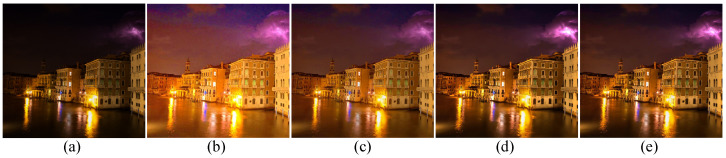
Ablation study for components of the CLAR model. (**a**) Input, (**b**) CLAR without $S_{x}{∥\nabla_{x}L∥}_{F}^{2}+S_{y}{∥\nabla_{y}L∥}_{F}^{2}$, (**c**) CLAR without $T_{x}{∥\nabla_{x}R∥}_{F}^{2}+T_{y}{∥\nabla_{y}R∥}_{F}^{2}$, (**d**) CLAR without $\sum_{i}{∥R_{i}\left(R\right)∥}_{*}$, (**e**) CLAR.

In Equation (7), the CLAR model contains four components, namely, the reconstructed term, the illumination smoothing term, the reflectance detail-preserving term, and the low-rank approximation term. To demonstrate the function of the components of the model, the subjective results of the ablation experiments are shown in Figure 12. In Figure 12b, the result generated by the CLAR without the illumination smoothing term tends to be overly sharp and color-distorted. In Figure 12c, the result generated by the CLAR without the reflectance detail-preserving term is blurry. By comparing Figure 12d,e, the low-rank approximation term could suppress the noise and improve the quality of the image. In addition to a subjective evaluation, the quantitative evaluations are demonstrated in Table 1. From Table 1, the CLAR consisting of each component ranks first.

### 4.7. Time-Consuming Evaluation

In order to compare the computing time of the proposed method and the conventional methods, the time-consuming evaluation is demonstrated in Table 2. For a fair comparison, the computational time was calculated by averaging the process time of ten images, which were resized to 960×720. As demonstrated in Table 2, the CLAR takes more time than the compared methods, but the results generated by the CLAR achieve satisfactory qualitative and quantitative effects. The increase in the computation time can be attributed to the fact that each additional regularization term leads to a significant increase in the amount of data to be processed.

## 5. Conclusions

In this paper, we proposed a constraint low-rank approximation Retinex (CLAR) model to enhance low-light images. Considering the noise mixed in the low-light image, the model is combined with exponential relative total variation constraints and low-rank prior. The constraints and low-rank prior ensure the piece-wise smooth illumination component and noise-free reflectance component. In addition, the alternating direction method of multipliers (ADMM) algorithm was utilized to solve the complexity problem. Comparative experiment results demonstrate that the proposed CLAR model achieves a compelling performance compared with the state-of-the-art methods.

## Figures and Tables

**Table 1:** Quantitative evaluation in terms of PSNR ↑ and SSIM ↑ metric for ablation test.

Component Ablation	PSNR ↑	SSIM ↑
CLAR without $S_{x}{∥\nabla_{x}L∥}_{F}^{2}+S_{y}{∥\nabla_{y}L∥}_{F}^{2}$	12.5327	0.3086
CLAR without $T_{x}{∥\nabla_{x}R∥}_{F}^{2}+T_{y}{∥\nabla_{y}R∥}_{F}^{2}$	18.8950	0.5091
CLAR without $\sum_{i}{∥R_{i}\left(R\right)∥}_{*}$	19.1743	0.5881
CLAR	19.6521	0.6078

**Table 2:** Comparison of time cost (in seconds).

Method	HE [33]	SSR [18]	MSRCR [34]	CVC [35]
Time	2.07	2.17	6.67	0.79
Method	Dong [36]	LIME [28]	Jiep [37]	Ours
Time	2.62	14.11	13.43	35.60

## Data Availability

Not applicable.
